# Corneal Confocal Microscopy (CCM) in Patients With Central Neurodegenerative Disease

**DOI:** 10.7759/cureus.116756

**Published:** 2026-09-22

**Authors:** Ayesha Malik, Maryam Ferdousi, Shazli Azmi, Rayaz A Malik

**Affiliations:** 1 Ophthalmology, Manchester Royal Infirmary, Manchester, GBR; 2 Research, University of Manchester, Manchester, GBR; 3 Endocrinology and Diabetes, Manchester Royal Infirmary, Manchester, GBR; 4 Medicine, Weill Cornell Medical College, Doha, QAT

**Keywords:** corneal confocal microscopy imaging, dementia, medical device technology development and assessment, neurodegeneration, parkinsons disease

## Abstract

Corneal confocal microscopy (CCM) is a rapid, non-invasive ophthalmic imaging technique that enables detailed visualization and quantification of small corneal nerve fibres. Although initially developed for the assessment of diabetic neuropathy, its application has broadened substantially, with increasing evidence supporting its value in a range of central neurodegenerative diseases. This narrative review synthesizes current findings on the use of CCM in dementia, multiple sclerosis, stroke, burning mouth syndrome, amyotrophic lateral sclerosis, trigeminal neuralgia, migraine and Parkinson’s disease.

Across these conditions, CCM consistently identifies measurable alterations in corneal nerve fibre density, length, branching and morphology, as well as changes in corneal immune cell populations. These structural abnormalities frequently correlate with disease severity, functional decline and clinical progression. In some disorders, CCM demonstrates diagnostic performance comparable to or exceeding existing biomarkers, and offers distinct advantages as a non-invasive, repeatable and objective measure that can detect early or subclinical neurodegeneration.

Corneal confocal microscopy also shows potential as a prognostic and monitoring tool, particularly in conditions with limited reliable biomarkers. It may help stratify risk, track therapeutic response, and provide surrogate endpoints for clinical trials. Despite these strengths, several limitations remain, including modest sample sizes in some studies, variability in image interpretation, and difficulties differentiating between neurodegenerative conditions with overlapping patterns of nerve loss. Emerging automated analysis methods and artificial intelligence-based algorithms may help address these challenges by improving accuracy, reproducibility, and scalability.

Overall, CCM represents a promising technique that bridges ophthalmology and neurology. A growing body of literature supports its utility across multiple neurodegenerative diseases, and further large-scale, longitudinal studies may help establish its role within routine clinical assessment and research.

## Introduction and background

Corneal confocal microscopy (CCM) has been extensively studied in large cohorts of subjects with diabetes, a range of other peripheral neuropathies, and increasingly in central neurodegenerative diseases. It is a rapid, non-invasive imaging technique that allows objective quantification of nerve degeneration and regeneration with high reproducibility and moderate to high sensitivity and specificity, enabling diagnosis, stratification of severity, and monitoring progression of peripheral neuropathy. It also has considerable potential as a surrogate endpoint in clinical trials of new therapies for the treatment of neuropathy [1]. This narrative review explores the increasing body of evidence showing that CCM has utility in a number of central neurodegenerative diseases.

A narrative literature search was conducted primarily using PubMed and Google Scholar to identify studies evaluating corneal confocal microscopy (CCM) in neurodegenerative disease. Search terms included combinations of “corneal confocal microscopy”, “corneal nerve fibre”, “neurodegeneration”, and conditions including dementia, multiple sclerosis, stroke, burning mouth syndrome, amyotrophic lateral sclerosis, trigeminal neuralgia, migraine, and Parkinson’s disease. Relevant studies published up to August 2026 were considered, with priority given to human studies evaluating CCM-derived nerve or immune-cell parameters, clinical correlations, diagnostic or prognostic utility, and longitudinal outcomes. Reference lists of key articles and reviews were also screened for additional relevant studies. As this was a narrative review, no formal risk-of-bias assessment or meta-analysis was undertaken.

## Review

Corneal nerves

Over the past several decades, the significance of evaluating corneal nerve morphology for assessing small fibre neuropathy has grown exponentially. Multiple studies have shown the evolving role of CCM in evaluating corneal nerve morphology as an objective marker of small nerve fibre damage in patients with peripheral neuropathies (PNs) [1-10].

The cornea is a highly specialised tissue and is the most densely innervated tissue in the body, with between 7000 and 16000 nerve fibres per mm2 - 300 to 600 times greater than the number of nerve fibres in skin [11]. The majority of innervation to the cornea is nociceptive; however, corneal nerves also respond to temperature and touch, as well as aiding in tear production and the corneal reflex - all of which aid in the maintenance of the functional and structural integrity of the eye through regulation of epithelial proliferation and wound healing in response to disease and trauma [12].

Corneal nerves consist of large diameter (6 µm) myelinated Aδ nerves mediating mechanical stimuli and small diameter (2-4 µm) unmyelinated C fibres mediating thermal and chemical stimuli [13]. These unmyelinated C fibres release neurotrophic factors such as nerve growth factor (NGF) in response to inflammation and aid in the corneal wound healing process as well as sustaining the normal cornea [12]. Corneal nerves are mainly derived from the ophthalmic division of the trigeminal nerve through the maxillary and anterior ciliary nerves. The limbus and peripheral corneal innervation also contain autonomic sympathetic ciliary nerves derived from the superior cervical ganglion [5,14].

Although the cornea is anatomically peripheral, alterations within the corneal sub-basal nerve plexus may reflect broader neurodegenerative and neuroinflammatory processes. Many disorders traditionally classified as diseases of the central nervous system (CNS) are increasingly recognised to involve widespread axonal degeneration, mitochondrial dysfunction and immune activation beyond the CNS. The sub-basal nerve plexus represents a readily accessible population of small sensory nerve fibres that can be visualised directly and repeatedly in vivo using CCM as potential surrogate markers of systemic neural integrity. Across multiple peripheral and central neurological disorders, abnormalities in corneal nerve morphology have been associated with established measures of disease burden, including structural neuroimaging, cognitive impairment, motor dysfunction and neurological disability. These observations support the concept that corneal nerve alterations may act as surrogate markers of neurodegeneration. The precise biological mechanisms linking corneal nerve changes to peripheral and central nervous system pathology remain incompletely understood.

Corneal confocal microscopy (CCM)

In vivo corneal confocal microscopy (IVCCM) is a non-invasive in vivo technique with a magnification of ~680 times, enabling rapid examination of the living cornea and quantitative assessment of corneal nerves. IVCCM was initially used to detect early nerve damage in diabetic neuropathy [15-17] and in the repair of corneal nerves after pancreas transplantation in patients with type 1 diabetes [18]. Additionally, using the first-generation IVCCM (Tomey ConfoScan, Tomey Corporation, Aichi, Japan), Tavakoli et al. identified small nerve fibre damage in patients with Fabry disease [19] and idiopathic small fibre neuropathy [20]. Subsequently, the second-generation IVCCM (Heidelberg HRT-III, Heidelberg Engineering GmbH, Heidelberg, Germany) was used to identify nerve fibre damage in hereditary neuropathies, e.g., type IV or V hereditary sensory and autonomic neuropathy (HSAN) [21], autoimmune peripheral neuropathy [22], non-length-dependent SFN [23], and chemotherapy-associated peripheral sensory neuropathy [24]. Most studies have assessed morphological changes in the corneal sub-basal nerve plexus; only a few have quantified changes to stromal nerves [5].

The deployment of corneal confocal microscopy (CCM) can be described as a four-stage process comprising preparation, imaging, analysis, and interpretation. Prior to the examination, the patient’s eyes are anesthetized and prepared using topical anesthetic drops and gel, which ensures adequate uninterrupted contact between the microscope lens and the cornea. The microscope is equipped with a specialized lens capable of capturing corneal nerve images, which is connected to a computer to enable image acquisition, storage, and analysis (Figure 1). The microscope objective is gently applied to the cornea, and images are obtained through rapid confocal scanning to acquire multiple non-overlapping, high-quality images of the central cornea (Figure 2). These images are then analysed using dedicated CCM software (CCMetrics and ACCMetrics by the Early Neuropathy Assessment (ENA) Group at Weill Cornell Medicine - Qatar and the University of Manchester), which quantifies a range of parameters to assess corneal nerve morphology (Figure 3). The results of this analysis are interpreted to provide clinically meaningful information regarding corneal nerve degeneration and regeneration.

**Figure 1:**
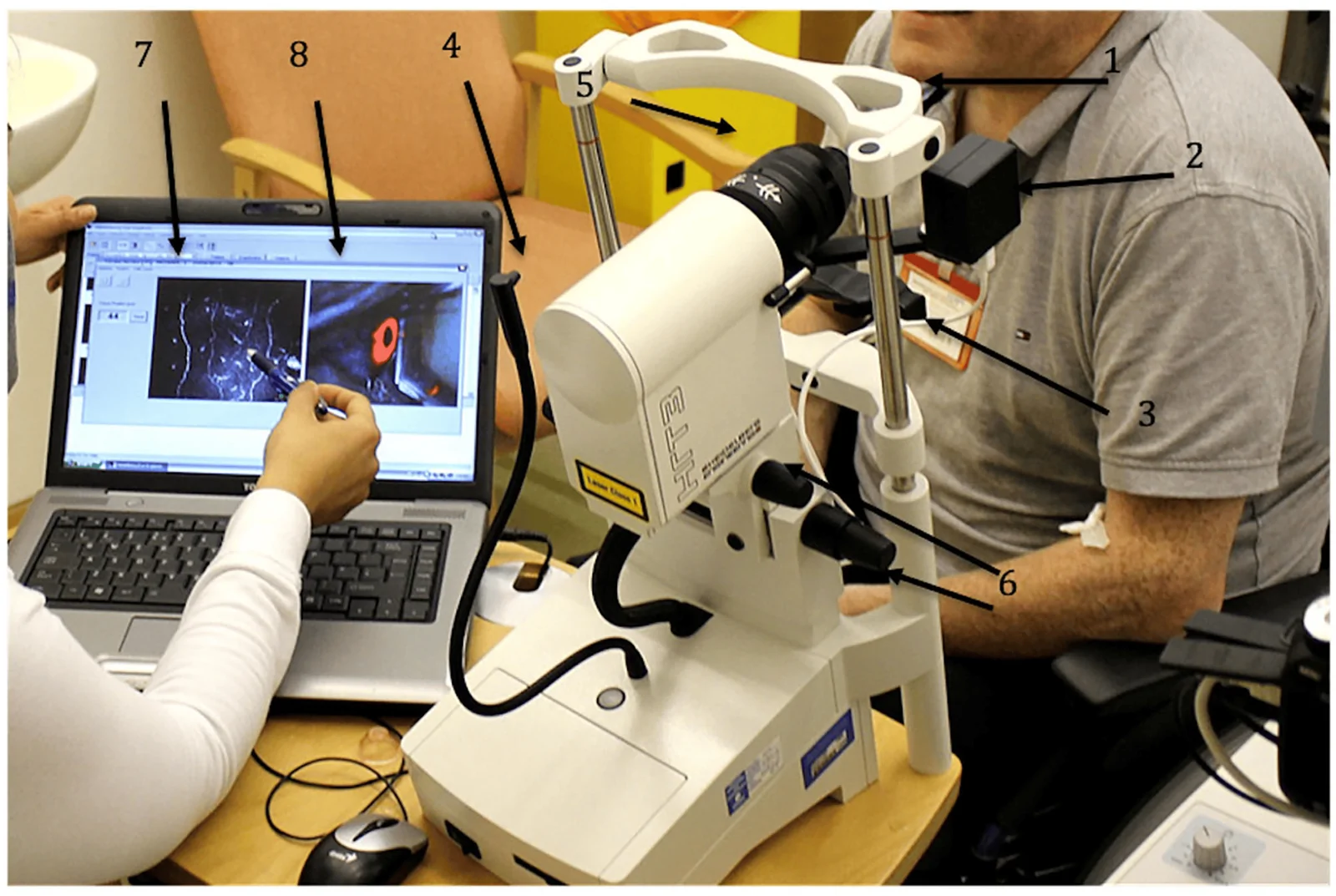
HRT III-RCM corneal confocal microscopy 1 – forehead bar, 2 – CCD camera, 3 – chin rest, 4 – fixation target, 5 – corneal module, 6 – knobs to align the CCM, 7 – CCM live image, 8 – CCD camera live image. Source: Petropoulos et al. [25]. Image available under Creative Commons Attribution 4.0 International license (CC BY 4.0). HRT III-RCM: Heidelberg Retina Tomograph III Rostock Corneal Module (Heidelberg Engineering GmbH, Heidelberg, Germany); CCD: charge-coupled device; CCM: corneal confocal microscopy

**Figure 2:**
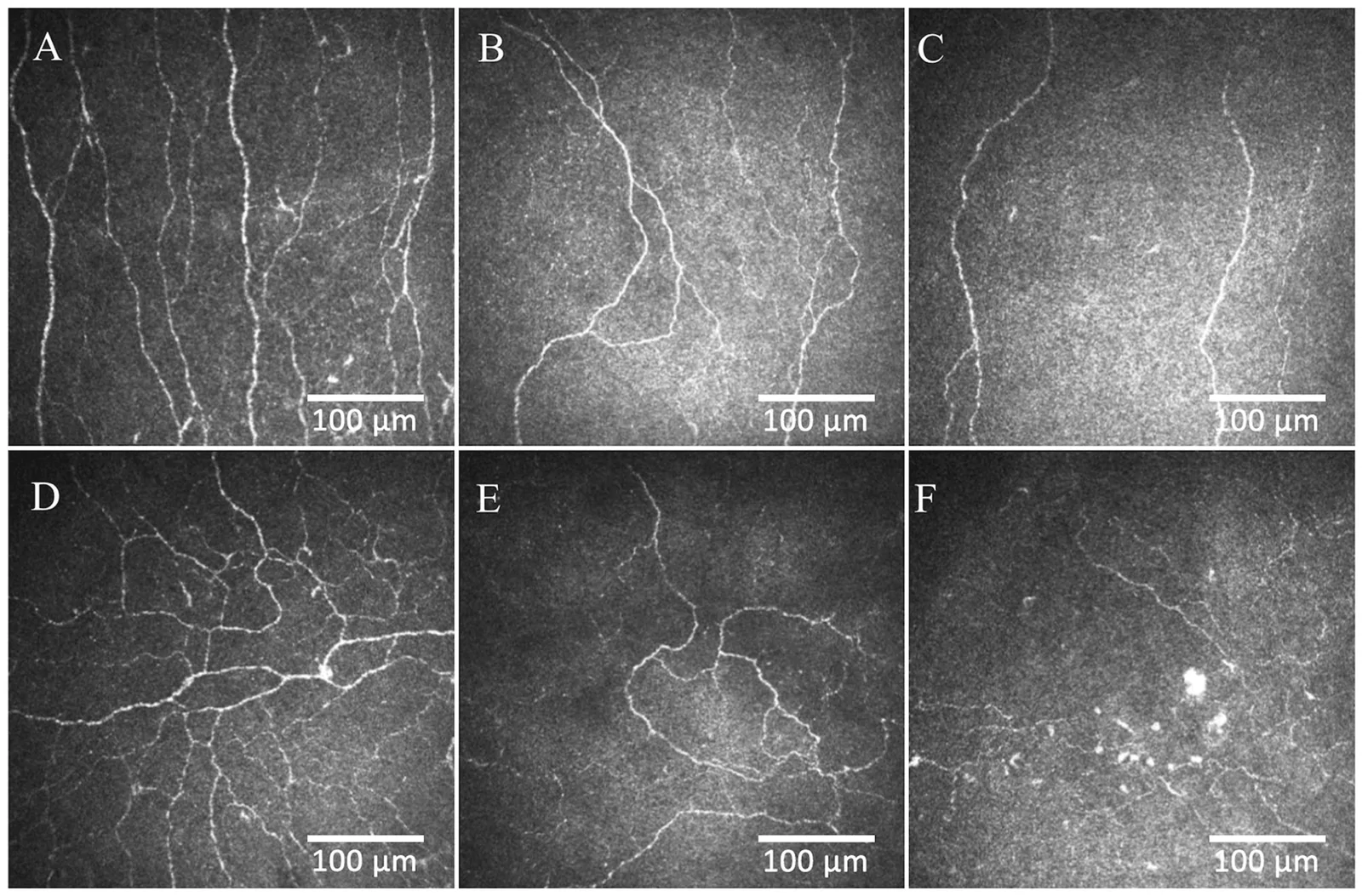
Primary CCM images taken from the central and inferior whorl region of the cornea Corneal confocal microscopy (CCM) images of the central cornea and inferior whorl in a healthy control (A, D) and in patients with painless (B, E) and painful (C, F) diabetic neuropathy. Source: Petropoulos et al. [25]. Image under Creative Commons Attribution License. Image available under Creative Commons Attribution 4.0 International license (CC BY 4.0).

**Figure 3:**
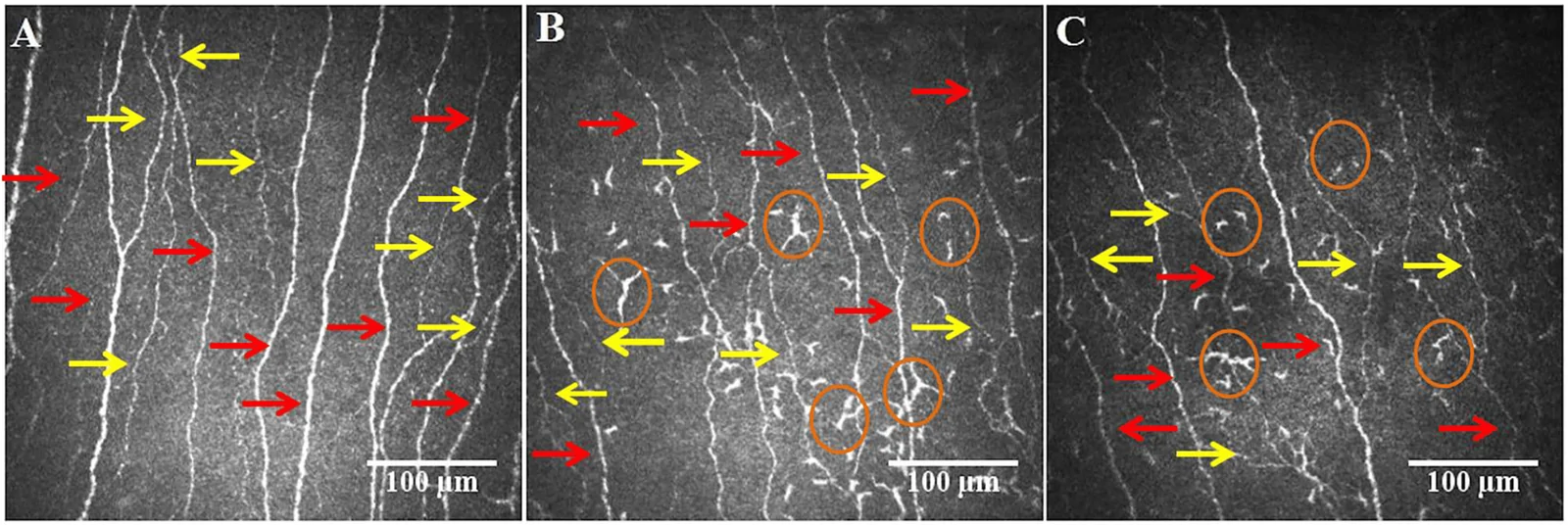
Various CCM images of the central cornea showing increased dendritic cells (circles), loss of main nerves (red arrows) and nerve branches (yellow arrow). Corneal confocal microscopy (CCM) images of the central cornea in a healthy control (A), post-COVID-19 patient without (B) and with (C) long-COVID, showing a loss of main nerves (red arrows) and branches (yellow arrows) in the patient with long-COVID and an increase in dendritic cells (circles) in patients with and without long-COVID. Source: Petropoulos et al [25]. Image available under Creative Commons Attribution 4.0 International license (CC BY 4.0).

Parameters used to quantify corneal sub-basal nerve morphology include nerve fiber and branch density, length, width, reflectivity, beading frequency, and tortuosity. The most common parameters include corneal nerve fiber density (CNFD), defined as the total number of nerves per square millimetre (fibers/mm2); corneal nerve branch density (CNBD), the number of branches originating from the main nerves per square millimetre of the cornea (branches/mm2); and corneal nerve fiber length (CNFL), defined as the total length of all the nerve fibers and branches per square millimetre (mm/mm2). CNBD has been proposed to be a viable marker of nerve regeneration as it captures new branches that sprout from the main nerves during nerve regeneration [26].

Corneal nerve parameters may be quantified manually, semi-automatically, or using fully automated analysis software. Manual analysis allows detailed, accurate assessment of nerve morphology but is time-consuming, operator-dependent, and requires expertise, which may introduce inter-observer variability. Automated platforms such as ACCMetrics were developed to provide faster and more reproducible quantification of the main parameters, e.g., CNFD, CNBD, and CNFL. More recently, artificial intelligence and deep-learning approaches have extended automated analysis beyond conventional nerve tracing, enabling the assessment of more complex morphological patterns and image-based classification. These developments may improve scalability and reproducibility; however, their clinical utility remains dependent on robust external validation and standardized image acquisition.

Corneal confocal microscopy (CCM) in dementia

Dementia is a progressive neurodegenerative condition encompassing Alzheimer’s disease (AD), vascular dementia, Lewy body dementia, and frontotemporal dementia, with prevalence increasing substantially with age [27]. Because cognitive decline occurs along a continuum from normal cognition to mild cognitive impairment (MCI) and dementia, identifying early transition points remains challenging [28,29].

Al-Janahi et al. (2020) compared CCM with medial temporal lobe atrophy (MTA) in patients with MCI and dementia and demonstrated significant corneal nerve loss in both groups compared with cognitively healthy controls, whereas MTA was predominantly observed in dementia. Corneal nerve measures showed greater diagnostic accuracy than MTA for distinguishing MCI from normal cognition and comparable performance in dementia [30]. Ponirakis et al. subsequently assessed 208 participants with no cognitive impairment, MCI, and dementia using Montreal Cognitive Assessment (MoCA), MRI brain volumetry, and CCM. CCM demonstrated greater discriminatory ability than MRI brain volumetry for distinguishing MCI from normal cognition, while both performed similarly in distinguishing dementia from controls. Corneal nerve abnormalities were also associated with poorer cognitive function after adjustment for confounding factors [31].

Further studies have shown that greater cortical and subcortical ischaemic burden is associated with more severe corneal nerve loss in MCI and dementia [32], while reduced corneal nerve measures have also been linked to declining cognitive function and reduced functional independence [33]. Collectively, these findings support CCM as a promising complementary biomarker for detecting and monitoring neurodegeneration in people with cognitive impairment.

More recently, Gundogan et al. (2024) demonstrated significant reductions in CNFL, CNFD, and CNBD in patients with AD compared with age- and sex-matched healthy controls, providing further evidence of sub-basal corneal nerve degeneration specifically in Alzheimer’s disease [34]. In addition to neuronal changes, Ponirakis et al. (2025) [35] demonstrated abnormalities in corneal endothelial cell morphology in MCI and dementia. In a cohort of 114 participants, CNFD, CNBD, and CNFL were significantly lower in both MCI and dementia compared to no cognitive impairment (NCI), while corneal endothelial cell density was significantly lower only in patients with dementia. Lower endothelial cell density and increased endothelial cell area were also associated with reduced cortical grey-matter volume, suggesting that CCM may capture both neuronal and neurovascular alterations associated with cognitive impairment [35].

Greater corneal nerve loss has been demonstrated in individuals with both cortical and subcortical cerebral ischaemia compared to those with cortical ischaemia alone, suggesting an association between the burden of cerebral ischaemia and corneal neurodegeneration [32]. Corneal nerve loss has additionally been associated with both declining cognitive function and reduced functional independence [33]. Collectively, these findings have contributed to the emerging concept of “neuro-oculomics”, whereby quantitative imaging of corneal nerves may act as a biomarker of peripheral and central neurological diseases. A recent review of the broader term of corneal oculomics has highlighted the potential for artificial intelligence-based analysis to integrate complex corneal structural features to improve disease identification [36]. The current evidence in dementia has been derived predominantly from relatively small cross-sectional cohorts, and CCM should therefore be regarded as a promising complementary biomarker rather than an alternative to established clinical assessment, neuroimaging or disease-specific biomarkers. Larger, longitudinal studies are required to determine whether CCM can reliably identify individuals with MCI at greatest risk of progression to dementia and whether corneal nerve changes differ sufficiently between dementia subtypes to provide disease-specific information.

Corneal confocal microscopy in multiple sclerosis

Multiple sclerosis (MS) is an autoimmune disease affecting the central nervous system and characterised by demyelination, axonal degeneration, and inflammation [37]. Magnetic resonance imaging (MRI) plays an important role in the diagnosis and monitoring of progression of MS but is not approved as a surrogate endpoint in clinical trials [38]. Optic neuritis is the most common ocular manifestation of MS, and optical coherence tomography (OCT) has been suggested as a useful biomarker to monitor retinal nerve fibre loss by quantifying retinal nerve fibre layer (RNFL) thickness [39].

Multiple studies utilizing CCM have identified a significant reduction in CNFD, CNBD and CNFL [40-43] with no change in corneal nerve tortuosity (CNFT) [40]. Earlier studies also reported conflicting findings in Langerhans and dendritic cell density, with increased, unchanged, or reduced densities [42, 44], which may reflect differences in MS subtype and ongoing disease-modifying therapy. A 2025 systematic review and meta-analysis of 12 studies, including 485 patients with MS and 319 healthy controls, confirmed significant reductions in CNFD, CNFL, and CNBD. Dendritic cell density did not differ significantly, but was increased in patients with a disease duration of eight years or less, suggesting that immune-cell alterations may be particularly relevant earlier in the disease course [45]. A longitudinal study has demonstrated altered corneal immune-cell populations in both relapsing-remitting MS (RRMS) and secondary progressive MS (SPMS), supporting a role for CCM to monitor both neuroinflammation and neurodegeneration [46].

Importantly, corneal nerve abnormalities may occur early in MS, as reduced CNFL and CNBD have been demonstrated in patients with early RRMS, despite no significant reduction in retinal nerve fibre layer thickness [47]. Furthermore, a significant limitation of OCT is that RNFL thickness may increase during an acute episode of optic neuritis, which limits its utility to assess disease progression and to assess the effect of therapeutic intervention. A significant reduction in CCM parameters has been demonstrated in patients with and without previous optic neuritis [40,41], suggesting that CCM may provide information complementary to OCT. Studies have consistently demonstrated associations between corneal nerve loss and neurological disability in MS [41-44]. Mikolajczak et al. (2017) reported an inverse association between CNFD and disease severity [42], while Petropoulos et al. found an association between CNBD and neurological disability [40]. Petropoulos et al. (2021) also recently demonstrated a progressive reduction in CNFD and CNFL in clinically isolated syndrome, relapsing-remitting MS, and secondary progressive MS [41].

Longitudinal studies further support an association between CCM abnormalities and disease progression. Over two years, worsening Kurtzke Expanded Disability Status Scale (EDSS) was accompanied by reductions in CNFL, corneal nerve fibre area (CNFA), corneal nerve fibre width (CNFW), corneal total branch density, and RNFL thickness, with increased Langerhans cell (LC) density and changes in CNFD and CNFL correlated with changes in EDSS [43]. In a 2023 study, RRMS was associated with lower CNFD and RNFL thickness but higher CNBD, while patients with SPMS had lower CNFD, CNFL, and RNFL thickness. CNBD had the highest area under the receiver operating characteristic curve (AUROC) for identifying RRMS (0.99) and CNFD for SPMS (0.95). Further reductions in corneal nerve parameters were observed at follow-up, with greater corneal nerve loss in patients who developed new enhancing lesions on MRI or optic neuritis [44]. A growing body of evidence for the utility of CCM in MS is supported by two recent meta-analyses. Al-Salahat et al. (2025) included eight studies and 351 patients with MS and confirmed significant reductions in CNFD and CNFL and an association between CNFD, CNBD, and CNFL and the Multiple Sclerosis Severity Score [48]. A second 2025 meta-analysis similarly confirmed significant reductions in CNFD, CNFL, and CNBD, although considerable heterogeneity remained between studies [45]. More complex automated measures may also provide additional discriminatory information. Petropoulos et al. (2024) demonstrated that automated corneal nerve fibre length and nerve fractal dimension were significantly lower in MS and could be used to distinguish patients with MS from those with epilepsy moderately well, but this requires validation in larger cohorts [49]. Finally, a 2026 scoping review of 50 studies identified MS and Parkinson's disease to have the strongest evidence supporting CCM, with CNFD and CNFL emerging as the most consistently altered parameters [50]. Nevertheless, heterogeneity in image acquisition, analysis techniques and study populations remains an important limitation, and larger standardised longitudinal studies are required before CCM can be incorporated into routine clinical assessment of MS.

Corneal confocal microscopy in stroke

Corneal confocal microscopy has been used to identify corneal nerve fibre loss in patients with ischemic stroke. Khan et al. found a significant reduction in CNFD, CNBD, and CNFL in patients with an acute ischemic stroke compared to healthy controls [51, 52]. Of note, the reduction in CNFD, CNFL, and CNBD was found in stroke patients with normal glucose tolerance [51]. Indeed, there was only a modest correlation between corneal nerve loss and elevated HbA1c and triglycerides; thus, corneal nerve loss in stroke patients is only partially explained by risk factors for peripheral neuropathy. Furthermore, in patients with acute ischemic stroke, alterations in corneal endothelial cell morphology showed a modest correlation with loss of corneal nerves [52]. Abnormalities in corneal nerves and endothelial cells have also been demonstrated in patients with transient ischemic attack (TIA) and minor stroke [53], although these studies had relatively large sample sizes of 130 [52] and 146 [53].

In patients with recurrent ischemic stroke, CNFD was significantly lower and corneal nerve fibre tortuosity (CNFT) was significantly higher than in patients with a first stroke [54]. White matter hyperintensities (WMH) are found in patients at risk of stroke and those with stroke [55], and higher levels of homocysteine, a risk factor for stroke, correlated with WMH [56]. Corneal nerve loss is associated with WMH in patients with acute ischemic stroke [57]. This suggests that corneal nerve loss is important in identifying patients at risk of stroke or recurrent stroke and in assessing the benefits of risk factor reduction [57]. These studies provide support for the utility of CCM in determining the risk of acute and recurrent ischemic stroke.

Corneal confocal microscopy in burning mouth syndrome

Burning mouth syndrome (BMS) is characterised by burning sensation/pain or dysesthesia of the oral mucosa, most commonly the tongue, without a causative or identifiable lesion. The incidence is higher amongst women between 50-60 years of age, and daily pain can seriously diminish the quality of life of patients with BMS [58]. CCM has been used to identify neurodegeneration, characterised by a lower CNFD and CNFL and inflammation evidenced by increased LC density in patients with BMS [59]. This supports the hypothesis that the pain in BMS is neuropathic [60] and CCM could be utilized instead of a tongue biopsy to identify underlying nerve damage [61].

Corneal confocal microscopy in amyotrophic lateral sclerosis

Amyotrophic lateral sclerosis (ALS), also known as Lou Gehrig’s disease or motor neurone disease (MND), is a neurodegenerative condition characterised by progressive loss of both upper (UMN) and lower (LMN) motor neurons in the brain and spinal cord. Fu et al. used CCM to show significant corneal nerve loss, especially in the inferior whorl region, which was associated with the severity of disease [62]. In a previous study, a reduction in CNFD and CNFL and an increase in CNBD and CNFT were associated with disease progression [63]. This finding is attributed to nerve degeneration and reinnervation, consistent with histopathological studies in skin biopsies from patients with ALS, which found comparable dual processes in both the autonomic and cutaneous sensory nerves [63]. Ferrari et al. showed that corneal nerve damage correlated with the bulbar function disability score, suggesting a link between ALS and facial-onset sensory and motor neuropathy syndrome (FOSMN) [64].

More recent evidence has been less consistent, with a prospective case-control study using automated CCM analysis showing no significant differences in CNFD, CNFL, CNBD, or other corneal nerve parameters between patients with ALS and healthy controls, and no association with disease duration, severity, or rate of progression [65]. Another study found no difference in CNFD or CNFL, but demonstrated increased density of corneal dendritic cells and frequency of axonal spheroids in patients with ALS [66]. These conflicting findings, albeit in relatively small studies, question the role of CCM as a diagnostic or prognostic biomarker in ALS. Larger longitudinal studies are required to establish whether corneal nerve or immune-cell alterations occur at particular stages of ALS and whether they relate to disease progression.

Corneal confocal microscopy in trigeminal neuralgia

Trigeminal neuralgia (TN) is a chronic neuropathic pain disorder which is commonly underdiagnosed or misdiagnosed. It presents with unilateral shooting facial pain but can also present bilaterally. Currently, there is no objective test to diagnose TN, and it is primarily diagnosed from the patients’ medical history, symptoms, and findings on a neurological exam. Lee et al. demonstrated a marked reduction in CNFD and CNFL in patients with TN, although the study was limited by a small sample size of only 11 patients [67]. A further study evaluating patients following balloon compression of the trigeminal ganglion or microvascular decompression also demonstrated alterations in corneal innervation, although the relationship between surgical intervention and underlying disease-related nerve changes remains difficult to distinguish [68].

More recently, Aydoğan et al. evaluated 60 patients with unilateral TN and 60 matched healthy controls and demonstrated significant reductions in CNFD, CNFL and CNBD, alongside increased nerve tortuosity, fragmentation and focal nerve dropout [69]. Increased dendritic cell density and anterior stromal keratocyte activation were also observed, particularly in patients with longer disease duration, while posterior stromal and endothelial morphology remained relatively preserved. Disease duration was independently associated with greater sub-basal nerve loss and inflammatory changes. These findings strengthen the evidence that TN is associated with both neurodegenerative and inflammatory alterations within the cornea and suggest that CCM may provide an objective measure of trigeminal nerve involvement. However, further longitudinal studies are required to determine whether these changes reliably reflect disease severity or progression and to distinguish disease-related alterations from those induced by previous surgical treatment.

Corneal confocal microscopy in migraine

Migraine is a complex neurological disorder that commonly presents as a unilateral headache with associated sensory symptoms such as photophobia. A longstanding hypothesis of migraine involves both sensitisation and activation of the trigeminovascular system. Shen et al. found that in patients suffering from episodic migraines (EM), there was an increase in LCs**,** suggesting underlying inflammation but with no significant change in CNFD or CNFL [70]. In contrast, Kinard et al. showed a significant decrease in CNFD and CNFL with comparable CNBD and CNFT in patients with chronic migraine, compared to controls [71]. Shetty et al. demonstrated a significant decrease in CNFL, CNBD and total nerve branch density in patients with photophobia and migraine compared to those with migraine without photophobia [72]. More recently, Guldiken et al. showed a comparable reduction in CNFD, CNFL, branch density, fibre width and area in patients with episodic and chronic migraine, with and without aura compared to controls [73].

A 2024 systematic review of pain profiling in migraine found conflicting CCM results across studies, likely reflecting small sample sizes and limited characterisation of migraine phenotype [74]. However, a subsequent systematic review of eight studies and 370 patients demonstrated a reduction in CNFD, CNFL and CNBD in patients with chronic migraine, but the findings in episodic migraine were more heterogeneous [75]. There were significant reductions in automated CNFD, CNFL and CNBD in patients with migraine compared with healthy controls, irrespective of whether they were episodic or chronic and independent of visual hypersensitivity [76]. This suggests that corneal small nerve fibre abnormalities may represent a broader feature of migraine pathophysiology rather than being confined to particular clinical subtypes or sensory manifestations.

Collectively, these findings support involvement of the trigeminal and peripheral sensory systems in migraine and suggest that CCM may provide a useful research biomarker of small fibre damage. However, heterogeneity between studies, differences in migraine phenotype and imaging methodology, and the absence of established disease-specific thresholds currently limit its diagnostic application.

Corneal confocal microscopy in Parkinson’s disease

Parkinson’s disease (PD) is a progressive neurodegenerative disease that affects about 2-3 % of the population older than 65 years of age and is accompanied by motor and non-motor symptoms [77]. Motor symptoms include tremor, bradykinesia, rigidity, muscle cramps, and dystonia, whilst non-motor symptoms include fatigue, excessive daytime sleepiness (EDS), autonomic dysfunction, and pain [25]. According to Braak, neurodegeneration begins in the peripheral nervous system (PNS), with subsequent involvement of the central nervous system (CNS) corresponding to the major motor manifestations [78]. This hypothesis is supported by studies demonstrating cardiac denervation, which precedes the loss of dopaminergic neurons in the substantia nigra [79]. Thus, CCM may be useful in screening, especially as skin biopsies have been used in the diagnosis of PD.

Several studies have demonstrated small nerve fibre degeneration in patients with PD [6, 80]. A significant reduction in corneal total branch density and nerve fibre area has been shown in patients with PD compared to healthy controls [81-83]. Misra et al. (2017) demonstrated a 50% reduction in CNFD in patients with moderately severe PD compared to healthy subjects [80], while automated CCM analysis has identified significant corneal nerve loss in PD [8]. Furthermore, corneal nerve abnormalities have been associated with motor severity, cognitive dysfunction, and autonomic involvement [82,83]. Lower baseline CNFL has also been shown to predict greater worsening of motor disability over 12 months, suggesting potential prognostic value [9]. More recent evidence extends these observations to non-motor manifestations, with greater corneal nerve loss associated with cognitive impairment and pain severity in PD [84,85]. In the latter study, combined CNFD, CNFL and corneal nerve fractal dimension discriminated patients with and without PD-related pain with an area under the curve (AUC) of 0.80 [85].

Earlier studies suggested that corneal nerve fibre loss did not differ significantly between PD motor subtypes; however, recent work has challenged this. A 2023 study demonstrated significantly greater reductions in CNFD, CNBD and CNFL in patients with postural instability and gait disturbance (PIGD) compared to tremor-dominant PD, with combined corneal nerve parameters differentiating these subtypes with an AUC of 0.832 [86]. Subsequent studies have demonstrated differences in corneal nerve morphology across PD phenotypes, suggesting that CCM may provide information beyond the presence of neurodegeneration alone [87]. 

Importantly, emerging evidence also suggests that CCM may aid differentiation between PD and other parkinsonian disorders. Corneal nerve loss has been demonstrated in multiple system atrophy (MSA) and progressive supranuclear palsy (PSP), although the extent of neurodegeneration varied between disorders [88]. In one study, CNFD and CNFL were significantly lower in MSA than in PD, suggesting potential utility in distinguishing these clinically overlapping conditions [89]. More recently, combined CNFD and CNBD differentiated idiopathic PD from secondary parkinsonism with an AUC of 0.924 [90]. These findings are particularly relevant because limited specificity has historically been considered an important limitation of CCM.

Overall, the evidence supporting CCM in PD, especially from China, is among the strongest across central neurodegenerative diseases, a finding reinforced by a recent scoping review in which PD and multiple sclerosis had the most substantial evidence base [50]. Nevertheless, important confounders remain. Diabetes may independently exacerbate corneal nerve loss in patients with PD, while treatments such as long-term amantadine can also induce corneal abnormalities [91]. CCM should therefore currently be considered a complementary biomarker rather than a standalone diagnostic test. Larger multicentre longitudinal studies are required to validate disease-specific thresholds and determine whether CCM can reliably contribute to differential diagnosis, prognostication and monitoring in routine clinical practice.

**Table 1:** Summary of CCM findings across neurodegenerative conditions. Key: ↓ = generally reduced; ↑ = generally increased; variable = inconsistent findings between studies. *Current evidence base represents a qualitative synthesis of the literature discussed in this narrative review and does not constitute formal evidence grading or risk-of-bias assessment. CCM: corneal confocal microscopy; CNFD: corneal nerve fibre density; CNFL: corneal nerve fibre length; CNBD: corneal nerve branch density; MCI: mild cognitive impairment; EDSS: Expanded Disability Status Scale; MSSS: Multiple Sclerosis Severity Score; OCT: optical coherence tomography; TIA: transient ischemic attack; CNFT: corneal nerve fibre tortuosity; PD: Parkinson's disease; MSA: multiple system atrophy.

Condition	CNFD	CNFL	CNBD	Other CCM findings	Clinical associations	Potential clinical/research utility	Current evidence base*
Dementia / MCI	↓	↓	↓	Reduced endothelial cell density in dementia	Cognitive impairment, functional decline, cerebral ischaemic burden, reduced cortical grey-matter volume	Early detection of neurodegeneration to complement cognitive assessment and neuroimaging	Moderate
Multiple sclerosis	↓	↓	↓ / variable	Dendritic cell changes variable; changes may differ with disease duration and treatment	EDSS/MSSS, disease subtype, progression, enhancing lesions and optic neuritis	Assessment of axonal loss; longitudinal monitoring; complementing OCT	Substantial
Stroke / TIA	↓	↓	↓	↑ CNFT in recurrent stroke; corneal endothelial abnormalities	White-matter hyperintensity burden, recurrent stroke, cerebral small-vessel disease	Research marker of neurovascular injury and risk stratification; prognostic utility remains unconfirmed	Moderate
Burning mouth syndrome	↓	↓	No	↑ Langerhans cell density	Supports neuropathic and immune mechanisms	Objective assessment of small-fibre pathology; research biomarker	Limited
Amyotrophic lateral sclerosis	Variable / ↓	Variable / ↓	Variable / ↑	↑ dendritic cells and axonal spheroids; ↑ CNFT reported previously	Disease severity and bulbar dysfunction in some studies	Investigation of sensory/small-fibre involvement; diagnostic and prognostic value remains uncertain	Limited / conflicting
Trigeminal neuralgia	↓	↓	↓ in recent study	↑ tortuosity, fragmentation and focal nerve loss; ↑ dendritic cells and stromal keratocyte activation	Disease duration; trigeminal nerve involvement	Potential objective marker of trigeminal small-fibre pathology; postoperative effects remain a confounder	Limited but emerging
Migraine	↓ / variable	↓ / variable	↓ / variable	↑ Langerhans cells in some cohorts	Migraine phenotype, photophobia/visual hypersensitivity and chronicity, although findings are inconsistent	Research biomarker of trigeminal and small-fibre dysfunction	Low–moderate / heterogeneous
Parkinson’s disease	↓	↓ / variable	↓ / ↑ depending on disease stage/phenotype	Altered nerve area, branching and fractal measures	Motor severity, cognitive impairment, autonomic dysfunction, pain, motor subtype and disease progression	Prognostication, phenotyping and differentiation of PD from MSA, atypical and secondary parkinsonism	Substantial

Discussion

Corneal confocal microscopy has the potential to act as an imaging biomarker of neurodegeneration in most neurodegenerative diseases. Originally used to assess diabetic neuropathy, it has grown into a rapid, non-invasive way to assess peripheral neuropathies of multiple etiologies. CCM has a robust body of evidence supporting its utility to help in the diagnosis and prognosis, as well as an endpoint in clinical trials of neurodegenerative diseases.

Limitations and Barriers to Clinical Translation

Despite the growing evidence supporting CCM in many neurodegenerative diseases, several limitations currently restrict its wider clinical application. Much of the available literature is derived from relatively small, single-center and predominantly cross-sectional studies, limiting the generalisability of findings and the ability to determine whether CCM can reliably predict disease progression or treatment response. Differences in image acquisition, including the region of the cornea examined, number of images selected, and analysis method used, may also contribute to variability between studies. Manual analysis is time-consuming and subject to inter-observer variation, while automated and artificial intelligence-based approaches require further external validation before widespread clinical implementation.

A further limitation is the lack of disease specificity. Reductions in CNFD, CNFL and other corneal nerve parameters have been demonstrated across multiple peripheral and central neurological disorders, making it difficult to attribute an abnormal CCM result to a single underlying condition. Rather than acting as a standalone diagnostic test, CCM may therefore be most useful when interpreted alongside established clinical and imaging biomarkers. For example, in Parkinson’s disease, CCM findings could potentially complement neurological assessment, including Unified Parkinson’s Disease Rating Scale (UPDRS) measures, and dopaminergic imaging where clinically indicated. In stroke, corneal nerve loss has been associated with the presence and severity of white matter hyperintensities [48], supporting a potential role for CCM in exploring the relationship between small vessel disease, neurodegeneration and cerebrovascular risk.

Interpretation is further complicated by potential confounders including age, ethnicity, diabetes and other peripheral neuropathies, dry eye disease, contact lens use, corneal pathology, previous ocular surgery and systemic or topical treatments. Disease-modifying therapies may also influence corneal nerve and immune-cell parameters, as demonstrated in studies of multiple sclerosis. However, these issues can be resolved with the development of age- and race-standardized normative values. In addition to this, CCM can provide information beyond conventional nerve-density measurements. For example, assessment of corneal immune-cell morphology has demonstrated alterations in dendritic and Langerhans cell populations in MS, providing additional insight into the inflammatory component of disease [2,4]. Such cellular-level analysis may complement conventional nerve parameters and contribute to a broader assessment of neurodegenerative and neuroinflammatory change.

There is currently no universally accepted disease-specific threshold for abnormal CCM findings, and large normative datasets accounting for demographic and ocular variables remain limited. Similarly, minimum clinically important differences for longitudinal changes in corneal nerve parameters have not been established across most neurological conditions. The cost and specialist infrastructure required for current CCM systems may also limit access outside specialist ophthalmic and research centres. Formal health economics studies will therefore be required to determine whether the potential benefits of earlier detection, longitudinal monitoring, and clinical-trial applications translate into cost-effectiveness.

Overall, larger multicenter longitudinal studies using standardized acquisition and analysis protocols are required to define disease-specific reference ranges, establish clinically meaningful changes over time, and determine how CCM should be integrated alongside existing neurological, imaging, and laboratory biomarkers.

Several of these limitations, particularly inter-observer variability, time-consuming manual analysis, and limited disease specificity, can be addressed through advances in automated image analysis and artificial intelligence.

Artificial Intelligence and Future Directions

Automated image-analysis methods have been developed to quantify conventional parameters such as CNFD, CNFL, and CNBD more rapidly and reproducibly. More recently, artificial intelligence (AI)-based deep-learning approaches have extended beyond simple nerve segmentation and quantification towards direct image classification and disease discrimination. Deep-learning algorithms have demonstrated performance comparable to manual analysis for quantifying corneal nerve morphology and may outperform conventional automated approaches in selected settings [92].

These developments form part of the emerging field of “oculomics”, in which ophthalmic imaging is used to identify biomarkers of systemic disease. Although initially applied predominantly to retinal imaging, the concept has increasingly been extended to the cornea, with recent work describing “corneal oculomics” as the integration of quantitative corneal imaging and AI to derive information about systemic and neurological health [36][93]. 

This approach may be particularly relevant to CCM because conventional measures of corneal nerve loss occur across multiple neurological disorders, thereby limiting disease specificity. However, recent deep-learning studies suggest that more complex image features may help address this limitation. In 2026, Rabah et al. developed NeuropathAI, an interpretable deep-learning multiclass classification system using CCM images to differentiate diabetic peripheral neuropathy, chemotherapy-induced peripheral neuropathy, chronic inflammatory demyelinating polyneuropathy, and HIV-associated sensory neuropathy. The model achieved an overall area under the receiver operating characteristic curve of 96.75%, with high disease-specific discrimination [94]. Although these findings were derived from peripheral neuropathies rather than central neurodegenerative disorders, they provide proof-of-concept that AI may identify disease-specific patterns within CCM images that are not captured by conventional nerve-density and length measurements alone.

Artificial intelligence may therefore improve the scalability, reproducibility, and discriminatory potential of CCM and facilitate integration of corneal imaging with other clinical, imaging, and molecular biomarkers. However, important limitations remain. Deep-learning systems require large, well-curated, and representative datasets, and their performance must be validated across different populations, imaging devices, and clinical settings. Algorithmic bias, limited external validation, and the interpretability of complex models also remain important barriers to clinical implementation. Consequently, AI-enhanced CCM should currently be regarded as an emerging research tool rather than an established diagnostic platform, with prospective multicentre validation required before its role in neurological practice can be defined. 

## Conclusions

Corneal confocal microscopy has evolved from an ophthalmic imaging technique utilized by a few ophthalmologists into a promising tool for assessing neural and inflammatory changes across a range of neurological and neurodegenerative disorders. The evidence reviewed here demonstrates recurring alterations in corneal nerve morphology and immune-cell populations, with particularly strong and increasingly consistent findings in Parkinson’s disease and multiple sclerosis. Associations with motor disability, cognitive impairment, disease severity, and longitudinal progression further support the potential of CCM as a clinically meaningful biomarker rather than simply an imaging finding.

The wider concept of “oculomics” reflects the increasing recognition that the eye can provide important information about systemic and neurological health. As automated image analysis and artificial intelligence continue to develop, CCM may become more reproducible, scalable, and capable of identifying complex disease-specific patterns that are not captured by conventional nerve-density measurements alone. These advances may help address some of the current limitations of CCM, including inter-observer variability and limited disease specificity.

Corneal confocal microscopy is unlikely to replace established neurological assessment, MRI, OCT, or disease-specific biomarkers, but it has considerable potential to complement them by enabling rapid, non-invasive, and repeatable quantification of neural and inflammatory change. Its future value may lie particularly in earlier identification of neurodegeneration, longitudinal monitoring, patient stratification, and the assessment of therapeutic response in clinical trials. Further multicentre longitudinal studies, standardized acquisition and analysis protocols, and disease-specific validation are required before routine clinical integration. Ultimately, the growing evidence base suggests that the eye may prove to be more than a window into systemic disease, instead becoming an increasingly valuable tool through which neurological disease can be detected, monitored, and better understood.
